# Biological Sound vs. Anthropogenic Noise: Assessment of Behavioural Changes in *Scyliorhinus canicula* Exposed to Boats Noise

**DOI:** 10.3390/ani11010174

**Published:** 2021-01-13

**Authors:** Giovanni de Vincenzi, Primo Micarelli, Salvatore Viola, Gaspare Buffa, Virginia Sciacca, Vincenzo Maccarrone, Valentina Corrias, Francesca Romana Reinero, Cristina Giacoma, Francesco Filiciotto

**Affiliations:** 1 Consiglio Nazionale delle Ricerche—Istituto per le Risorse Biologiche e le Biotecnologie Marine, Messina (IRBIM-CNR)—Spianata S. Raineri, 86, 98122 Messina (ME), Italy; virg.sciacca@gmail.com (V.S.); francesco.filiciotto@cnr.it (F.F.); 2 Dipartimento di Scienze della Vita e Biologia dei Sistemi, Università degli Studi di Torino, 10123 Torino (TO), Italy; cristina.giacoma@unito.it; 3 eConscience—Art of Soundscape, No-Profit Organization, via Provinciale 610, 90046 Monreale (PA), Italy; 4 Centro Studi Squali—Istituto Scientifico presso Aquarium Mondo Marino—Loc. Valpiana, 58024 Massa Marittima (GR), Italy; primo.micarelli@gmail.com; 5 Istituto Nazionale di Fisica Nucleare (INFN)—Laboratori Nazionali del Sud, 95100 Catania (CT), Italy; sviola@ins.infn.it; 6 Consiglio Nazionale delle Ricerche—Istituto per lo studio degli impatti Antropici e Sostenibilità in ambiente marino, Capo Granitola (IAS-CNR)—Via del Mare, 3, 91021 T.G. Campobello di Mazara (TP), Italy; gaspare.buffa@cnr.it (G.B.); vincenzo.maccarrone@cnr.it (V.M.); 7 Dipartimento di Scienze Marine, Ecologia e Biologia—Università degli Studi della Tuscia—Largo delle Università, 01100 Viterbo (VT), Italy; valcorrias@gmail.com; 8 Dipartimento di Ecologia—Università della Calabria—Via Pietro Bucci, 87036 Rende (CS), Italy; fr.reinero@gmail.com

**Keywords:** small-spotted catshark, biological sounds, anthropogenic noise, signal/noise ratio

## Abstract

**Simple Summary:**

To date there is not much information regard the role that sounds may play in the life of elasmobranchs. This gains particular importance if we consider the current understanding about noise pollution at sea. In fact, in the past few years, the effects of anthropogenic noise on marine fauna have received increasing attentions considering the plethora of repercussions deriving from the expansion of this type of pollution. Here, we exposed small-spotted catshark specimens kept in an aquarium, to different acoustic conditions to analyse the possible changes in swimming behaviour. Four different acoustic conditions consisted of biological sounds and anthropogenic noises. Moreover, the amplitude levels were differentiated among them, to analyse the effects caused by different signal-to-noise ratios. The results highlighted both a tendency of the animals to increase the overall time spent swimming and to avoid the noisiest section of the aquarium when subjected to higher amplitude levels of noise.

**Abstract:**

Despite the growing interest in human-made noise effects on marine wildlife, few studies have investigated the potential role of underwater noise on elasmobranch species. In this study, twelve specimens of small-spotted catshark (*Scyliorhinus canicula*) were exposed to biological and anthropogenic sounds in order to assess their behavioural changes in response to prey acoustic stimuli and to different amplitude levels of shipping noise. The sharks, individually held in aquariums, were exposed to four experimental acoustic conditions characterized by different spectral (Hz) components and amplitude (dB re 1 µPa) levels. The swimming behaviour and spatial distribution of sharks were observed. The results highlighted significant differences in swimming time and in the spatial use of the aquarium among the experimental conditions. When the amplitude levels of biological sources were higher than those of anthropogenic sources, the sharks’ swimming behaviour was concentrated in the bottom sections of the aquarium; when the amplitude levels of anthropogenic sources were higher than biological ones, the specimens increased the time spent swimming. Moreover, their spatial distribution highlighted a tendency to occupy the least noisy sections of the aquarium. In conclusion, this study highlighted that anthropogenic noise is able to affect behaviour of catshark specimens and the impact depends on acoustic amplitude levels.

## 1. Introduction

To understand a species’ key bioecological activities in aquatic environments, as in terrestrial environments, it is important to examine the role of hearing. There is good evidence that sound is used by marine organisms in different contexts, such as alarm calls warning of danger, orientation cues, territory defense, searching for prey, mating behaviour, and parental care [1,2,3,4,5,6,7,8]. Within the marine environment, during the past few decades, the acoustic sense has been subjected to high levels of underwater noise pollution [9], especially from shipping vessels [10,11,12], leading to significant alterations in both animals and habitats [9,13,14,15,16]. Consequently, anthropogenic noise appears in the United Nations Convention on the Law of the Sea (UNCLOS) and in European legislation such as the Marine Strategy Framework Directive 56/2008 CE.

Noise pollution can drive acoustic interference compromising the ability to effectively perceive acoustic information between aquatic organisms [17]. This phenomenon can also affect the acoustic relations between species and their environment, interfering with ecological strategies [18,19,20,21]. In this regard, it must be noted that non-vocal species, may also take advantage of the sounds produced by other species to gather useful information. For example, Myrberg et al. [22], when studying the behavioural response of free-ranging sharks to low frequency pulsed sounds, hypothesised that these species monitor the sounds of struggling fish in order to locate and capture their prey.

The hearing sense of elasmobranchs has received little attention in the last thirty years [23,24,25,26,27,28,29], and therefore the overall hearing abilities of these animals remain largely unknown [30]. Some studies have shown that elasmobranchs have an acoustic sensitivity threshold between 20 and 1500 Hz (optimum, 40–600 Hz) [31] and they are mainly attracted by low intermittent frequencies [22]. The abovementioned studies suggest that the auditory sensitivity of these cartilaginous fishes falls within the frequency range where human-made noise is of highest amplitude [10]. Nevertheless, at present there are few studies investigating the role that soundscape may have on sharks’ environmental perception [32] and, consequently, on the potential capacity of noise pollution to affect elasmobranch’s behaviour.

Behavioural responses such as avoidance, escape, and motility, may reduce or eliminate the probability of death defending the organism against hostile conditions [33]. In this context, Mauro et al. [34], analysed behavioural responses of juvenile *Sparus aurata* exposed to low frequency noise, and reported significant changes in group dispersion, motility, and swimming height. Therefore, the study of behavioural perturbations can play an important role in improving our understanding of animal responses to arising adverse conditions [13,35,36,37], such as in a noisy environment [38,39,40,41].

The small-spotted catshark, *Scyliorhinus canicula* (Linnaeus, 1758), is a small shark present in the Northeast Atlantic Ocean and in the Mediterranean Sea, feeding on small bottom-dwelling invertebrates (crustacean, gastropods, cephalopods, and worms) and fish. Moreover, *S. canicula* has been considered to be an interesting laboratory study animal since Wintrebert [42]; it is the first species of Chondrichthyes for which the complete mitochondrial genome sequence was obtained [43] and it is considered to be an “emerging chondrichthyan model” [44]. These characteristics, together with its abundance and its easy maintenance in a controlled environment, have made this species an important model for the scientific community [45,46,47,48,49].

Although there are no studies specifically focused on the inner ear of *S. canicula*, several considerations can be derived from the study by Evangelista et al. [50] on the external morphology of the membranous inner ear of elasmobranchs; specifically, on the results coming from other benthic demersal species (e.g., brownbanded bamboo shark, *Chiloscyllium punctatum* and Port Jackson shark, *Heterodontus portjacksoni*). The morphological characteristics of the hearing organ of these sharks, have part of their semicircular canals bound to the dorsal surface of the saccular chamber, giving the ears a triangular appearance, and relatively small saccular organs; a characteristic belonging to non-raptorial foragers, feeding mainly upon marine invertebrates and with the highest sensitivity at low frequencies [51].

However, there is a lack of published data from a behavioural point of view, such as the expected responses to acoustic stimuli or responses to unfavourable conditions.

The aim of the present study was to analyse the potential effects caused by the presence of anthropogenic noise on behavioural responses of *S. canicula* specimens subjected to different acoustic experimental conditions. More specifically, we attempted to answer the following questions: (1) Can biological sounds from potential prey organisms affect the behaviour of *S. canicula*? (2) Do sharks react differently to different noise conditions, i.e., can the intensity of a shark’s behavioural response be signal/noise dependent?

## 2. Materials and Methods

### 2.1. Animal Housing

The study was conducted at the Centro Studi Squali, Aquarium, Massa Marittima (SW Tuscany, Italy), where twenty-two small-spotted catsharks were relocated to one indoor rectangular glass aquarium (2.5 m in length, 1 m in width, and 1.5 m in height) for a month-long acclimation period, after being captured between Elba Island and the Sardinia by trawling activity. During this period, the sharks were maintained under natural photoperiods and were fed with frozen molluscs and shrimp ad libitum until two days before the start of the experiment.

The holding and experimental aquariums reproduced a Tyrrhenian sandy circalittoral environment and were equipped with an independent flow-through seawater system. A salinity of 36 ± 1 ppt (mean ± SD) and a temperature 17.6 ± 0.8 °C (mean ± SD) were maintained during the entire study period.

Six females and six males of 228 ± 23 g in weight (mean ± SD) and 39.3 ± 0.8 cm in length (mean ± SD), were used in the experiment (for a total of twelve specimens).

### 2.2. Experimental Design

The experimental study was carried out in a rectangular aquarium (4 m in length, 1 m in width, and 1.5 m in height) with 2 cm thick glass walls, where the animals were exposed to one of four acoustic experimental conditions. In order to create the acoustic experimental conditions, two preliminary acoustic tracks were audio-created: the first, (A) anthropogenic, consisted of hydrofoils, ferries, fishing, and recreational boats noises; the second, (B) biological, consisted of sea urchins grazing, snapping shrimps, and teleost vocalizing (*Sciaena umbra*) acoustic emissions (more details on the audio mixing processes are given in Section 2.3).

**Figure 1:**
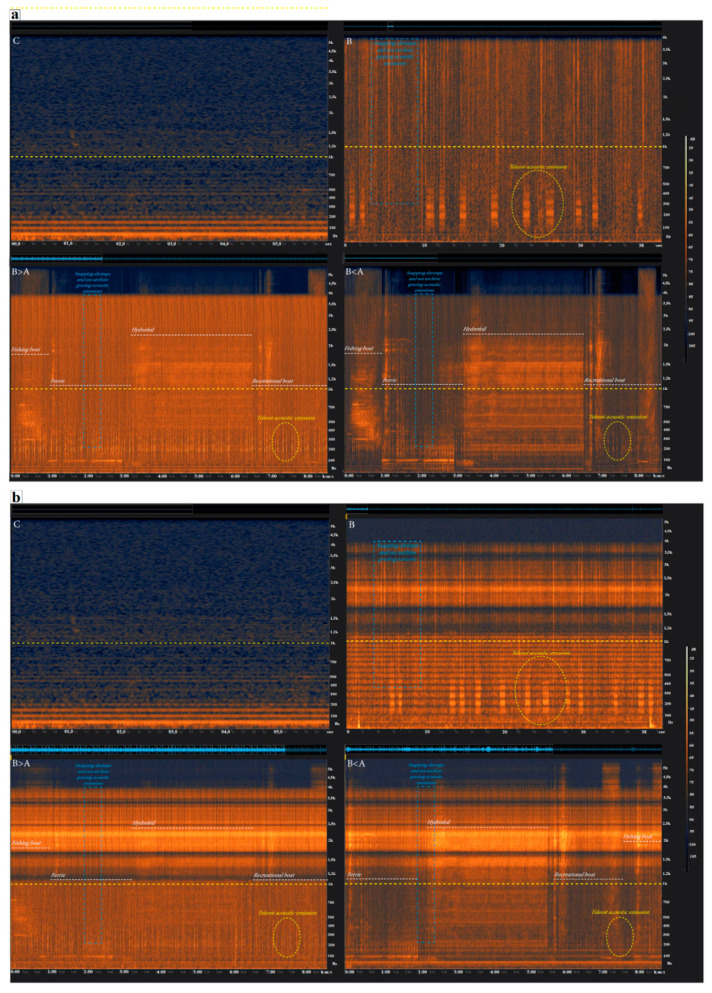
(**a**) Spectrogram (FFT 2048, Hanning window, frequency scale linear) of the four acoustic experimental conditions, showing the different spectral features; (**b**) Spectrogram (FFT 2048, Hanning window, frequency scale linear) of the four acoustic experimental conditions recorded inside the experimental aquarium, showing the different spectral characteristics. The yellow dotted line represents the upper hearing sensitivity threshold limit of elasmobranchs benthic demersal species [25,50]. C, control condition; B, biological condition; B > A, biological > anthropogenic condition; B < A, biological < anthropogenic condition.

Through a combined use of these two preliminary acoustic tracks, the acoustic experimental conditions (Figure 1a) were created as follows:

- (B) biological acoustic condition: A ten-minute audio file recreating the main acoustic components of a marine rocky soundscape, using signals from snapping shrimps, sea urchins grazing, and siniferous fish.
- (B > A) biological > anthropogenic acoustic condition: A ten-minute audio file where the abovementioned track was mixed with another ten-minute audio file, resembling an intense shipping traffic marine area. Hydrofoils, recreational boats, ferries, and fishing boat noises were used to achieve this target. In this condition, the biological sounds were 6 dB higher above the anthropogenic noise.
- (B < A) biological < anthropogenic acoustic condition: A ten-minute audio file similar to the abovementioned “biological > anthropogenic” track but, in this case, the biological sounds were 6 dB lower than anthropogenic noise.
- (C) Control condition: Characterized only by low-level background noise of the experimental aquarium.

Three replicates were conducted for all the acoustic experimental conditions. The study’s experimental design is reported in Table 1. In total, twelve ten-minute trials were run back-to-back in the experimental aquarium, adopting a random sequence of experimental conditions, during which the sharks’ swimming behaviours were monitored and recorded.

The trials started after 10 min of habituation and each specimen was used in only one trial to satisfy the postulate of experimental independence.

The animal husbandry and experimentation protocols were reviewed and approved in accordance with the Directive 2010/63/EU.

### 2.3. Acquisition, Editing and Projection of Acoustic Stimuli

A calibrated hydrophone (model AS-1, Aquarian Audio, Washington, DC, USA) with a flat sensitivity of −209 dB re 1 V/μPa up to 100 kHz was employed to collect all the acoustic recordings utilised for the audio-mixing processes. The hydrophone was used with a preamplifier (model PA-4, Aquarian Audio, Washington, DC, USA) with a gain value of +26 dB and connected to a digital acquisition board (model UMC204HD, Behringer, Willich, Germany) managed by the SeaPro software. Signals were acquired at 44 100 samples s^−1^ at 16 bits and visualized by the Rx5 software (iZotope, Cambridge, MA, USA). The biological sounds of grazing sea urchins and snapping shrimps were recorded in a marine rocky area called Cala Pisana, whereas the teleosts were recorded in Capo Grecale (both sites in Lampedusa Island, Italy). The anthropogenic noise sources were recorded in an area near the SW coasts of Sicily (coordinates 37°38.39′ N–12°35.19′ E) according to the following two criteria: the equal distance between hydrophone and boat (about 300 m), and the absence of other boats within a radius of 8 km.

After the recording phase, the files were subsampled at 11 025 samples s^−1^ and two preliminary tracks were created using the sound-editing software Rx5. First, the “biological” (B) track was edited mixing in the *S. umbra* emissions, with the recordings characterized by the presence of sea urchin grazing and snapping shrimp sounds.

Then, the “anthropogenic” (A) track was edited by mixing the recordings of four different types of boats, i.e., a fishing vessel, a ferry, a hydrofoil, and a recreational boat.

**Figure 2:**
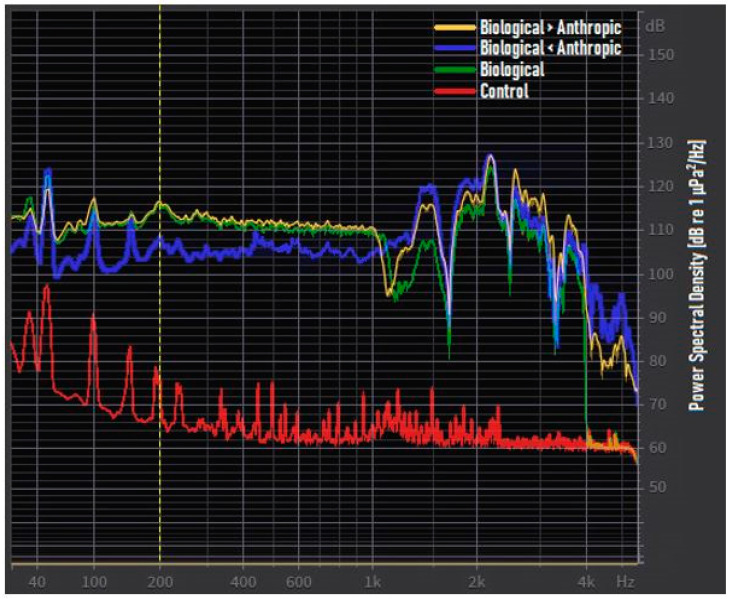
Averaged power spectrum of the three audio-created acoustic tracks and the aquarium’s background noise. The power spectral density (PSD) is expressed in dB re 1 μPa2/Hz versus the logarithmic frequency scale expressed in Hz. The yellow dotted line represents the limit below which the sound speaker has a limited frequency response.

The combined use of these two preliminary acoustic tracks allowed for the creation of the “biological > anthropogenic” (B > A) and “biological < anthropogenic” (B < A) tracks. For these passages, the sound pressure levels (SPLs, dB re 1 μPa) of the “biological” and “anthropogenic” files were calculated using MATLAB (MathWorks, Inc., United States). At this point, it was possible to raise or lower, using the gain tool of Rx5 software, the “biological” file by +/− 6dB with respect to the “anthropogenic” file to create the “Biological > anthropogenic” and “biological < anthropogenic” acoustic tracks, respectively. As a reference point, the amplitude peak was fixed between 80 and 200 Hz, due to the impulsive signals of *S. umbra* [52]. The amplitude levels were established between 100 and 140 dB re 1 μPa [23]. Figure 2 shows the power spectral density (PSD, dB re 1 μPa2/Hz) of the aquarium’s background noise and the audio-created acoustic tracks acquired in the experimental aquarium. Table 2 shows the mean sound pressure levels (SPL, dB re 1 μPa).

An underwater speaker (model LL916C, Lubell, Columbus, OH, USA) was used to emit the acoustic stimuli inside the experimental tank. The signal came through the stereo output of a PC connected to a power amplifier (model NX3000D, Behringer, Willich, Germany).

### 2.4. Behavioural and Audio Monitoring System and Analysis

Two observers, working concurrently, manually recorded the behavioural data, adopting the methodology defined by Altmann [53] for focal animal sampling. In order to validate the correctness and conformity of the data collected manually, all the experimental tests were video recorded with a camera (Skynet Italia s.r.l.) placed in front of the aquarium and linked to a 4 channel LCD DVR with a 7 inch screen, H.264.

**Figure 3:**
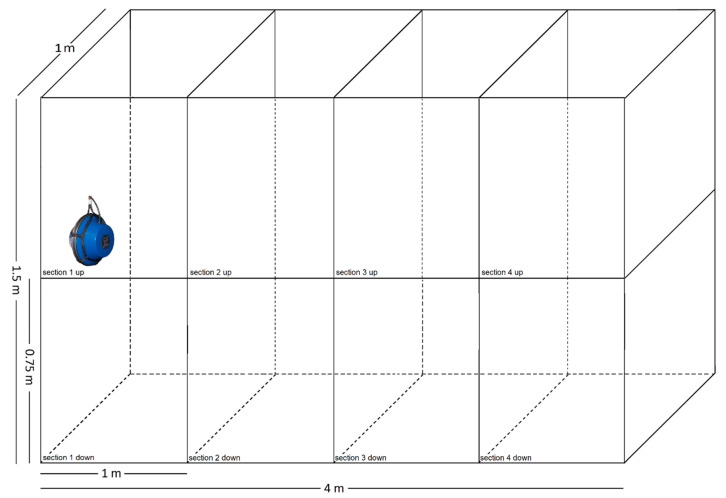
Subdivisions of the aquarium in cells and sectors. Section Up, constituted by 1, 2, 3, and 4 up cells. Section Down, constituted by 1, 2, 3, and 4 down cells. Section Left, constituted by 1–2 up and 1–2 down cells. Section Right, constituted by 3–4 up and 3–4 down cells. On the left wall, the location of the underwater sound speaker is presented.

The experimental aquarium was subdivided into eight cells of equal size, 100 cm long and 75 cm high (see Figure 3), in order to easily monitor the sharks’ behaviours (swimming time and spatial occupancy). During the trials, one observer assessed (1) overall swimming time during the entire experimental session for each specimen, while another observer recorded (2) the aquarium spatial occupancy in terms of time spent swimming by the animals in each cell.

After the data collection phase, the results of the parameters measured in real time by the two observers were compared to the video recordings that confirmed the reliability of the data collected manually with a percent agreement of 100%.

The laboratory setup for audio monitoring and recording was installed at 2 m distance from the experimental aquarium in order to avoid disturbing the catsharks during the experimental sessions. A calibrated hydrophone (model AS-1, Aquarian Audio, Washington, DC, USA) was used to record the baseline noise of the aquarium and three acoustic condition files (Figure 1b). Signals from hydrophone were acquired using a Zoom H6 handy recorder (Zoom Corporation, Tokyo, Japan) through the preamplifier (model PA-4, Aquarian Audio, Washington, DC, USA) with a gain value of +26 dB. The input impedance of the preamplifier was 2.2 MΩ, its output impedance was 50 Ω. The hydrophone was placed at 0.75 m depth in the center of the aquarium and signals were acquired with a sample rate of 11 025 s^−1^ at 16 bits. Knowing the sensitivity of the hydrophone-preamplifier assembly and the input voltage corresponding to the nominal full scale of Zoom H6 recorder, it was possible to obtain calibrated measurements of the data SPLs in µPa.

Otherwise, in order to analyse the sound amplitude attenuation in the experimental aquaria, each of the three acoustic condition files was recorded in each of the eight cells. In all acoustic conditions, the mean sound pressure showed a gradual decrease, moving from the cells closer to the transducer to those that were further away.

### 2.5. Statistical Analysis

As far as the swimming behaviour is concerned, the time (s) specimens spent in each cell was calculated for each replicate belonging to the same experimental condition (B, B > A, B < A, and C). Then, the data gathered was tested for goodness-of-fit to normal distribution using the Chi-square test. Afterwards, observation of different distribution patterns in behavioural data was followed by the application of nonparametric tests to compare the different values obtained among the experimental conditions.

The Kruskal–Wallis test was used to determine the influence of the acoustic experimental condition on swimming time, whereas the Mann–Whitney U test was applied to assess the effect on spatial occupancy of the aquarium (up/down and left/right sections). Cluster heatmaps were used to show the density of cell occupancy in terms of time spent by sharks among the four acoustic experimental conditions.

Statistical analyses were conducted using the SPSS (IBM, Armonk, NY, USA) software package. *p*-Values of *p* < 0.05 were considered to be statistically significant.

## 3. Results

### 3.1. Overall Time Spent in Swimming

**Figure 4:**
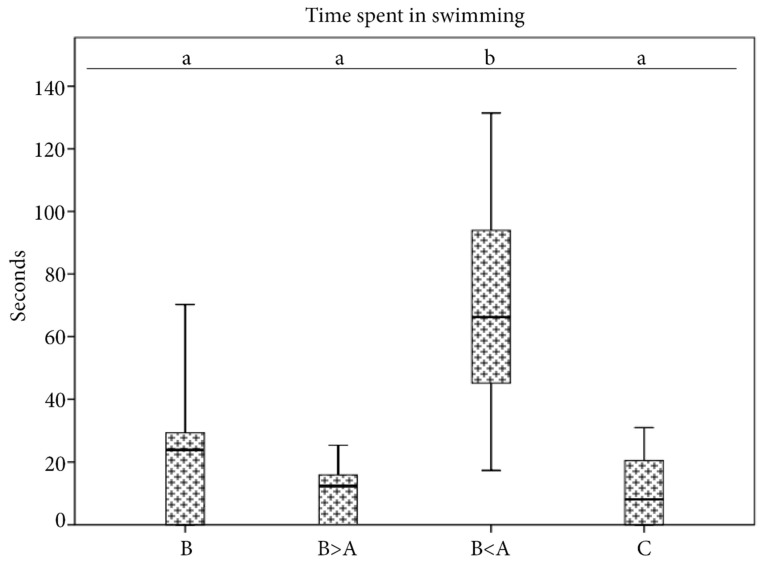
Values (median ± 25th–75th percentiles, whiskers ± 1st–99th percentiles) of the time spent in swimming assessed in *S. canicula* in the four acoustic experimental conditions. Different letters represent significant differences among the acoustic experimental conditions (*p* < 0.05).

Significant differences among the experimental acoustic conditions were shown by the statistical analysis considering the variable swimming time. The greatest increase in this behavioural parameter was shown for the specimens subjected to the “biological <anthropogenic” condition as compared with the other acoustic experimental conditions (Kruskal–Wallis test, *H* = 39.4, *N* = 96, df = 3, *p* = 0.0005). No significant differences were found among the other acoustic experimental conditions (Figure 4).

### 3.2. Spatial Occupancy of Aquarium

In terms of spatial occupancy, there were no significant differences in swimming time among cells.

**Figure 5:**
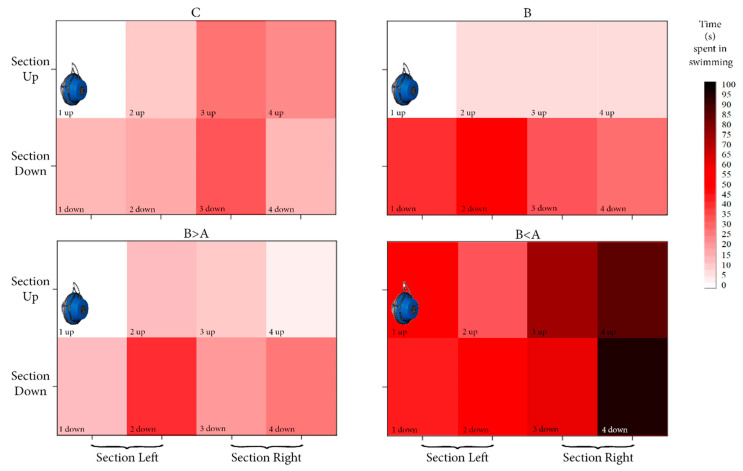
Cluster heatmap showing the spatial occupancy in terms of time spent in swimming (expressed in seconds) by the sharks among the aquarium cells. Section Up, constituted by 1, 2, 3, and 4 up cells. Section Down, constituted by 1, 2, 3, and 4 down cells. Section Left, constituted by 1–2 up and 1–2 down cells. Section Right, constituted by 3–4 up and 3–4 down cells. On the left wall, the location of the underwater sound speaker is presented. C, control condition; B: biological condition; B > A, biological > anthropogenic condition; B < A, biological < anthropogenic condition.

To highlight the spatial response with respect to the acoustic experimental conditions, we considered the differences in spatial occupancy between the up and down, left and right, sections of aquarium (see Figure 5 for the subdivision).

Specifically, in the “control” condition, the sharks showed a homogenous use of the space without significant differences in time spent between the up/down and between the left/right sections of the aquarium (Figure 5).

In the “biological” condition, they showed a heterogeneous use of the space; in fact, it is possible to observe a statistical difference (Mann–Whitney U test, U = 12.5, N1 = 12, N2 = 12, *p* = 0.05) in swimming time between the up and down sections (6.2% and 93.8% of the total time spent in swimming, respectively), but also a difference, although not statistically, in the left section with respect to the right section (65.1% and 34.9%, respectively) (Figure 5).

In the “biological > anthropogenic” condition, the specimens showed heterogeneous use of the space, highlighting a significant difference (Mann–Whitney U test, U = 35.5, N1 = 12, N2 = 12, *p* = 0.05) in swimming time between the up and down sections of the aquarium (about 16.8% and 83.2%, respectively) (Figure 5).

Finally, the sharks showed homogenous use of the space in the up and down sections in the “biological < anthropogenic” condition and made a different use of the left and the right sections (about 36.3% and 63.7%, respectively), although not statistically significant (Figure 5).

## 4. Discussion

### 4.1. Behavioural Response in the Control Condition

The results of the *S. canicula*’s swimming behaviour in the control condition highlighted a low mobility rate strategy of specimens. In fact, of the 1800 s analysed in the three replicates of this condition, the total amount of time spent moving was 392 s. This result was consistent with the information reported by Sims et al. [54], which showed a high value of resting for *S. canicula* specimens kept in laboratory (0.6 min h^−1^ spent active during daytime and 14.5 min h^−1^ spent active during nighttime). Nevertheless, the spatial distribution showed a homogenous use of the space, with no specific tendencies towards certain sections of the aquarium.

### 4.2. Behavioural Response in the Biological and Biological > Anthropogenic Conditions

The feeding habits of small-spotted catsharks are mainly adapted to preying on benthic species [55,56], and the analysis on stomach sampling showed a predilection for decapod crustaceans [57]. This means that the predatory behaviour of this species takes place in close contact with the substratum [58]. In this study, the “biological” file was constructed using the sounds emitted by shrimps, sea urchins, and fish (brown meagre), the main biophonies (see Pijanowski et al. [59] for a description of soundscape components) of the Mediterranean coastal environment [60].

The behavioural results of this study showed that the catsharks spent significantly more time moving in the down section of the aquarium as compared with the up section, and a propensity towards the down cells, which are closer to the transducer. Moreover, the acoustic results elucidated a gradual attenuation of sound amplitudes from the nearest to the farthest cells from the acoustic source.

The limited behavioural knowledge regarding this species does not permit an adequate explanation of the results. One interpretation could suggest that in *S. canicula*, the acoustic sense, in addition to the sense of electroreception and the sense of smell [57], might assist this species to better locate its prey. In every case, the different behavioural responses observed in the trials suggest a possible capacity of *S. canicula* in the distinction of our different acoustic stimulations and their amplitude changes. The experimental design of this study aimed at evaluating whether the same sound had different impacts on the catshark’s behaviour depending on the signal/noise amplitude ratio. Despite the reduced responsiveness of the sound speaker for frequencies below 200 Hz, the bandwidth between 200 and 1200 Hz was also acoustically reliable concerning the expected hearing abilities of *S. canicula* [25,50,51].

In the “biological > anthropogenic” condition, the amplitude of the biological signal was 6 dB higher than the noise and this condition did not seem to alter the specimens’ behaviour as compared with the “biological” acoustic condition. Otherwise, no avoidance behaviour, restlessness, and increased motility ascribable to acoustic noise pollution [35,37,61,62] were observed. From these results, it would appear that noise amplitude levels, when lower than biological sounds, do not operate as pollutants, based on the behaviour in *S. canicula* specimens in aquariums.

### 4.3. Behavioural Response in the “Biological < Anthropogenic” Condition

A very large number of marine organisms are exposed, on a daily basis, to moderate but widespread low-frequency noise produced by several anthropogenic activities such as vessels, seismic explorations, offshore wind farms, and other human activities [10,63].

This study analysed, for the first time, the effects of vessels noise on the swimming behaviour of of Carcharhiniformes sharks. The analysis of the sharks’ swimming time showed significantly increasing values in the specimens subjected to the “biological < anthropogenic” acoustic condition. This result is in agreement with other studies conducted on fish species showing restless responses and increased motility due to noise pollution [35,61,62]. In *Thunnus thynnus* specimens exposed to noise generated by boats, Sarà et al. [36] underlined an increase in several swimming behaviours, such as position changes in the water column. In that study, the authors also hypothesised that such behavioural response indicated avoidance and escape behaviours from the source. In the present study, the effects of noise also appeared to have a significant influence on the catshark’s swimming behaviour. In fact, the specimens involved in this experimental condition showed the biggest increase in swimming time, and 63% of this value was spatially distributed in the section farthest from the transducer (the right section of the aquarium, see Figure 5). Despite some earlier studies indicating that low frequency sounds elicit an avoidance reaction [64,65,66], there are not many studies on avoidance and escape as possible responses to noise in fishes [37,67]. For example, Berthe and Lecchini [35], on evaluating the escape behaviour due to boat noise on white-spotted eagle rays (*Aetobatus ocellatus*), showed that such a pollutant might elicit this behaviour in 60% of the samples examined.

Therefore, this study would seem to indicate some degree of choice by catsharks for being as far away from the sound source as possible, suggesting the role of anthropogenic noise as a disturbing phenomenon. The results, in fact, showed that noise pollution has aroused both apparent escape behaviour, as the catsharks’ swimming time increased, and apparent avoidance behaviour, since the sharks spent more time (>60%) in the aquarium sections farthest from the noise source.

High responsiveness may have a negative impact on this species with a low mobility rate strategy, affecting the metabolic costs, and then the energy cost of self-maintenance or resting metabolic rate (RMR) [68]. Moreover, from a natural environment perspective, the avoidance behaviour showed by the specimens could also represent a risk to carrying out key behaviours such as feeding or mating, thus, reducing available habitat use [33]; therefore, the survival capability of individuals, populations, and of the whole ecosystems could be compromised [63,69].

## 5. Conclusions

This study, for the first time, tried to clarify the effects of different acoustic conditions on sharks, by evaluating behavioural changes in relative, rather than absolute terms, of four acoustic experimental conditions, in a closed environment.

The results validate the concepts expressed by Ellison et al. [70] on the importance of a multiple contextual approach to study the impacts of noise, since multiple contextual factors can affect how animals respond to the noise exposure.

In light of these results, it seems that, in a close environment, noise could modify the behaviour of *S. canicula*, significantly increasing their swimming activity and changing their spatial distribution, as discussed in the “behavioural response in the biological < anthropogenic condition” caption. Despite the absence of a physiological analysis on the metabolic costs in this study, we know that a very large part of the fish energy budget increases when swimming activity increases [35,71,72]. In this condition, the evidence of increased swimming activity and the potentially associated metabolic costs could certainly compromise other key biological activities, as showed by other authors [73,74,75]. However, in order to expand the data available on the effects of noise pollution, further studies should also be conducted in an open natural environment where walls do not influence the acoustic field, such as in small aquariums.

Finally, in view of growing sea noise pollution levels and their potential effects on marine organisms, European legislation such as the Marine Strategy Framework Directive 56/2008 CE aims to achieve a good environmental status (GES) of marine waters through monitoring and mitigation actions of the phenomenon. Furthermore, as envisaged by the Maritime Spatial Planning (MSP) Directive, the ecosystem-based approach (Article 1 (3) 2008/56/EC) should be applied to ensure that anthropogenic pressures remain within the limits set for achieving the GES [76]. Within the next years, member states should adopt systems to assess the health status of the seas and set up any corrective actions.

The assessment of the impact of anthropogenic noise on wild marine life involves several difficulties, due to the high variability of the natural environments. Hence, experimental studies conducted in controlled conditions (i.e., tanks, mesocosms, and aquarium) to identify animal responses to anthropogenic sounds and the role of the acoustic signals in animal interactions, become of crucial importance. For these reasons, the marine management programs would benefit from including laboratory studies complemented by field monitoring activities (passive acoustic monitoring).

## Figures and Tables

**Table 1:** Schematic view of the experimental condition design.

Experimental Condition	Acoustic Features	N° Specimens Per Trial	N° Replicates	TOT Specimens Involved	TOT Trials
Control (C)	Low-level background noise of the experimental aquaria	1	3	3	3
Biological (B)	Acoustic file representing the main acoustic components of marine rocky soundscape	1	3	3	3
Biological minor of anthropogenic (B < A)	Acoustic file representing the main acoustic components of marine rocky soundscape mixed with the noise produced by the shipping traffic. The biological sounds were 6 dB less intense than the anthropogenic shipping traffic noise.	1	3	3	3
Biological major of anthropogenic (B > A)	Acoustic file representing the main acoustic components of marine rocky soundscape mixed with the noise produced by the shipping traffic. The biological sounds were 6 dB more intense than the anthropogenic shipping traffic noise.	1	3	3	3

**Table 2:** Mean, maximum, minimum, and standard deviation of experimental conditions sound pressure levels (SPLs) (dB re 1 μPa) recorded inside the experimental aquarium.

Experimental Acoustic Condition	SPL dB re 1 μPa Band 15 Hz–5.5 kHz	
Control	Mean	74.0
	Maximum	98.1
	Minimum	56.4
	SD	±11.6
Biological	Mean	107.2
	Maximum	129.1
	Minimum	71.0
	SD	±13.6
Biological > anthropogenic	Mean	107.0
	Maximum	129.0
	Minimum	74.3
	SD	±13.8
Biological < anthropogenic	Mean	101.0
	Maximum	126.0
	Minimum	56.3
	SD	±19.6
